# Machine learning plus optical flow: a simple and sensitive method to detect cardioactive drugs

**DOI:** 10.1038/srep11817

**Published:** 2015-07-03

**Authors:** Eugene K. Lee, Yosuke K. Kurokawa, Robin Tu, Steven C. George, Michelle Khine

**Affiliations:** 1 Department of Biomedical Engineering, University of California, Irvine, Irvine, CA 92697; 2 Department of Biomedical Engineering, Washington University in St. Louis, St. Louis, MO 63130; 3 Department of Statistics, California Polytechnic State University, San Luis Obispo, San Luis Obispo, CA 93410; 4 Department of Energy, Environment, and Chemical Engineering, Washington University in St. Louis, St. Louis, MO 63130; 5 Department of Chemical Engineering and Material Science, University of California, Irvine, Irvine, CA 92697.

## Abstract

Current preclinical screening methods do not adequately detect cardiotoxicity. Using human induced pluripotent stem cell-derived cardiomyocytes (iPS-CMs), more physiologically relevant preclinical or patient-specific screening to detect potential cardiotoxic effects of drug candidates may be possible. However, one of the persistent challenges for developing a high-throughput drug screening platform using iPS-CMs is the need to develop a simple and reliable method to measure key electrophysiological and contractile parameters. To address this need, we have developed a platform that combines machine learning paired with brightfield optical flow as a simple and robust tool that can automate the detection of cardiomyocyte drug effects. Using three cardioactive drugs of different mechanisms, including those with primarily electrophysiological effects, we demonstrate the general applicability of this screening method to detect subtle changes in cardiomyocyte contraction. Requiring only brightfield images of cardiomyocyte contractions, we detect changes in cardiomyocyte contraction comparable to – and even superior to – fluorescence readouts. This automated method serves as a widely applicable screening tool to characterize the effects of drugs on cardiomyocyte function.

The current state of drug development is costly and inefficient: only 1 out of 5,000 compounds available at the drug discovery stage will achieve Food & Drug Administration (FDA) approval, and the process takes approximately 14 years at a cost of 1.5 billion U.S. dollars^1^. A significant portion of this cost is attributed to withdrawal of drugs in clinical phases or post-FDA approval, 30% of which is from cardiotoxicity^2^. For example, the diuretic drug Cisapride’s undetected cardiotoxic effects resulted in 175 deaths and 386 cases of serious ventricular arrhythmia before it was removed from the market in 2000^3^.

Clearly current preclinical screening methods do not adequately detect cardiotoxicity. The advent of human induced pluripotent stem cell-derived cardiomyocytes (iPS-CMs) creates the possibility of a better *in vitro* model of the human myocardium for various applications including drug screening^4^,^5^,^6^. While current protocols result in iPS-CMs that are immature^7^, they do express several important phenotypic characteristics including key contractile and channel proteins^8^. However, a persistent challenge for developing a high-throughput drug screening platform using iPS-CMs is the need for a simple and reliable method to measure key electrophysiological and contractile properties.

Invasive methods, such as patch clamping, are traditionally limited to single cell analysis, and have proven difficult for high-throughput applications. Attempts to incorporate patch-clamping into high-throughput and commercial use are limited by cell membrane quality^9^. In addition, the instability of the seals prevents extended or longitudinal studies^10^,^11^. Fluorescence-based optical methods such as voltage and calcium sensitive dyes provide non-invasive means to observe electrophysiological properties of iPS-CMs^12^. However, these dyes can impact cell function, and therefore are not suitable for prolonged studies. Furthermore, both dyes and genetically encoded indicators are subject to photobleaching effects^13^,^14^. Microelectrode arrays (MEA) have high-throughput capabilities, but require a cluster of CMs for accurate electrical signals^15^,^16^. Similarly, impedance-based measurements offer non-invasive, high-throughput methods of drug screening, but are limited to monolayer cell cultures^17^,^18^.

Therefore, there remains a need to develop a high-throughput, sensitive, yet non-invasive detection platform for iPS-CMs. We have previously demonstrated a platform that utilizes brightfield images of iPS-CMs to measure drug effects on cardiac behavior (e.g. the positive chronotropic effects of isoprenaline)^19^. Optical flow analysis is performed on the images to generate vectors representative of cardiomyocyte motion. This inexpensive and non-invasive imaging method requires only the use of a brightfield microscope and camera, and is thus applicable to longitudinal studies in cell clusters, monolayers, and individual cells. To enhance this methodology, our current study pairs the brightfield optical flow method with a computational analysis method: supervised machine learning. Machine learning can evaluate multiple parameters simultaneously without *a priori* knowledge; therefore, it can discover unexpected relationships to potentially yield better detection. Furthermore, machine learning provides a singular quantitative index that summarizes the impact of multiple parameters, and thus simplifies the assessment of drug effects on cardiomyocytes. We hypothesize that combining machine learning with optical flow detection will produce an automated, high-throughput methodology that is more sensitive than fluorescence-based detection schemes to capture drug-induced effects on human iPS-CMs.

To test our hypothesis, we evaluated the iPS-CMs response to three cardioactive drug compounds with distinct, dissimilar effects: E-4031 (hERG K^+^ channel inhibitor), verapamil (L-type Ca^2+^ channel blocker), and blebbistatin (myosin-II inhibitor). The concentration range for each drug was selected based on the demonstrated window of cardioactive effects in previous studies^16^. To the best of our knowledge, we report for the first time the integration of optical flow with machine learning on iPS-CMs to detect drug induced cardioactive effects, and demonstrate superior performance to fluorescence-based methods.

## Results

### Brightfield and Fluorescence Signal Processing

**Figure 1:**
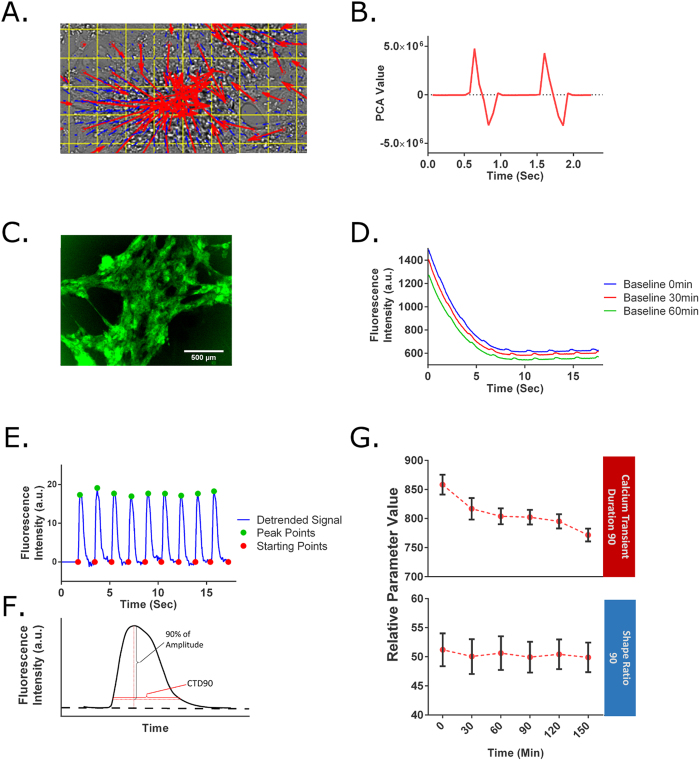
Data acquisition and analysis for GCaMP6 and brightfield signal. (**A**) For the brightfield method, images are processed by an optical flow algorithm to generate vectors that represent the motion of the iPS-CMs. The blue vector arrows represent the average motion of 20 × 20 pixel regions, while the red vector arrows represent 50 × 50 pixel regions. The convergence of the vector arrows toward a center visually represents an iPS-CM cluster in the contraction phase. (**B**) Based on the vectors, a contractile profile is derived from the 1^st^ PCA of the norm of the x- and y-vectors. Positive PCA values indicate contraction phase, while negative values signify the relaxation phase. (**C**) GFP signal produced by the contraction of cardiomyocytes derived from GCaMP6 iPS cells. (**D**) GCaMP6 signal recorded over time for 3 sequential acquisitions at baseline. The signals show a rapid photobleaching effect within the first 5 seconds, but also show a photobleaching effect between the different acquisitions. (**E**) The starting points and peaks of the calcium transients are identified using the detrended GCaMP6 signal. (**F**) A graphical explanation of CTD_90_. The SR_90_ is defined as the ratio of CTD_90_ to the amplitude of a given calcium transient. (**G**) A longitudinal experiment shows an artificial decrease in CTD_90_ due to photobleaching. This is corrected by normalizing by the amplitude in SR_90_.

For the brightfield analysis, optical flow was used to generate motion vectors representing iPS-CM contractions (Fig. 1a). Using principal component analysis (PCA), we summarized these motion vectors into a contractile profile that contains distinct peaks that represent the contraction (positive PCA values) and relaxation (negative PCA values) as seen in Fig. 1b.

For this study, we chose Support Vector Machine (SVM) as the machine learning algorithm. In SVM, a machine is trained with data to create an optimal model with generalizability. SVM classifies the data points into two groups (e.g. normal and abnormal cardiomyocyte behavior) by creating a decision boundary that separates the two groups. The model is evaluated by classifying unseen or withheld data. This evaluation yields an accuracy value (SVM accuracy) that reflects the effectiveness of the model. The percentage accuracy represents the ability to identify an effect (e.g. 98% represents out of 100 samples, the machine can correctly classify 98 of the instances). In the absence of cardioactive compounds, we would expect the accuracy of the machine learning to be random and therefore generate a SVM accuracy of approximately 50%. Implementation of SVM is described in detail in the Methods.

As a fluorescence-based method, we utilized an iPS cell line transduced with a genetically encoded fluorescent calcium indicator, GCaMP6. This method is similar to those found in industry for the high-throughput screening of drug-induced cardiotoxicity^20^,^21^,^22^. In this study, the raw GCaMP6 signal acquired over time shows a rapid decay due to photobleaching (Fig. 1d). The photobleaching artifact was eliminated by fitting a polynomial curve to the baseline of the signal, defined as the starting point of the calcium transient, and then normalizing the raw signal by the baseline value (Fig. 1e). In addition, the starting points were normalized to zero using linear interpolation. From this data, we calculated the beating rate and the calcium transient duration 90% (CTD_90_), defined as the duration of the fluorescent signal that was 90% below the peak amplitude of the transient (Fig. 1f). CTD_90_ is described in detail in the Methods.

In addition to the rapid photobleaching over a single acquisition time frame, we noted that the GCaMP6 signal also experiences a smaller photobleaching effect over multiple acquisitions (Fig. 1g). This loss of signal intensity caused artificial decreases in CTD_90_; this was corrected by normalizing CTD_90_ with the amplitude of the calcium transient signal. This corrected value is referred to as the Shape Ratio 90 (SR_90_), and serves as one of the two parameters analyzed for the GCaMP6 method (Fig. 1g). This effect was independent of time between acquisitions (Supplemental Fig. 2).

### Control experiments

**Figure 2:**
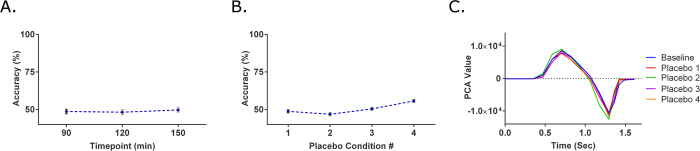
Analysis of control experiments: longitudinal and placebo experiment. (**A**) In the longitudinal experiment, behavior of iPS-CMs was monitored for an extended period of 150 minutes at 30 minute intervals after baseline measurements. When the machine was tasked of classifying between the first and last half of the timepoints, the SVM accuracies were approximately 50% as expected (n = 13). (**B**) To ensure the drug delivery process had no effects on iPS-CMs, at each placebo condition, 10 μL of media was added to the previous tested condition. The average SVM accuracy of all four placebo conditions was 50.42%, similar to that of the longitudinal experiments (n = 18). (**C**) Representative traces of the contractile profile from the brightfield analysis show no differences among tested conditions.

To ensure that the experimental setup (i.e. stage top incubator), time, and other external factors do not significantly impact the behavior of iPS-CMs, a longitudinal experiment was performed. iPS-CMs were recorded with both the GCaMP6 method and brightfield method for a total of 150 minutes at intervals of 30 minutes. In the analysis of the GCaMP6 signals, there were no changes in the beating rate and SR_90_ (Supplemental Fig. 3), indicating stability in the iPS-CM behavior during the length of the experiment. For the brightfield method, the SVM classification was performed by grouping the first three measurements (0, 30 and 60 minutes) as the baseline control in a similar fashion to the GCaMP6 analysis. The baseline control was then compared to the 90, 120 and 150 minute time points. Corresponding SVM accuracies of 48.64 ± 1.51, 48.22 ± 1.57, and 49.67 ± 1.41% were calculated (Fig. 2a), which are consistent with no longitudinal effect.

The purpose of the placebo experiment was to evaluate the drug delivery process and serve as the vehicle controls. In each placebo condition, 10 μL of RPMI/B-27 media, the vehicle for drug treatment, was added to the iPS-CMs, for a total of four placebo deliveries. Analysis with the GCaMP6 method indicated no change in beating rate and SR_90_ values when compared to the three baseline measurements taken prior to the placebo treatments (Supplemental Fig. 4). In the brightfield method, the SVM accuracies were 48.75 ± 1.01, 46.86 ± 1.19, 50.40 ± 0.98, and 55.67 ± 1.03% respectively for each of the placebo treatments (Fig. 2b), again consistent with no effect. Thus, the drug delivery process had no significant impact on the behavior of iPS-CMs.

### Response to E-4031

**Figure 3:**
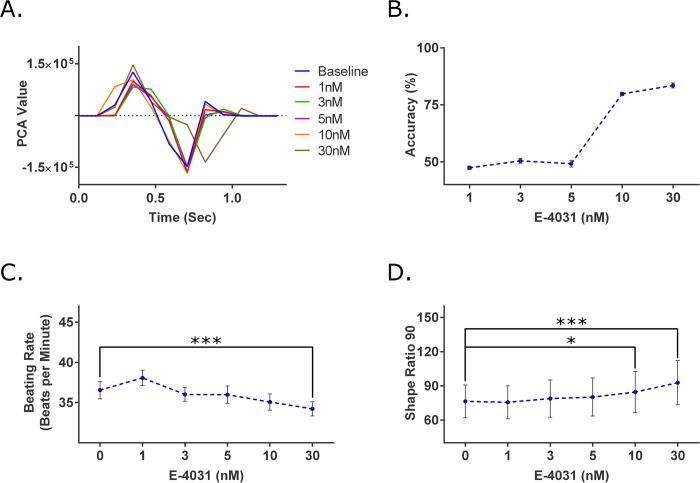
Analysis of E-4031, a hERG K^+^ channel blocker known to prolong the QT interval. (**A**) Representative traces of the contractile profile from the brightfield analysis show a pronounced contraction elongation at the highest tested concentration (30 nM). (**B**) At the concentrations of 1, 3, and 5 nM, the SVM accuracies were approximately 50%, similar to those of the control experiments. For the 10 and 30 nM concentrations, the SVM accuracies increased to 79.82 ± 0.77% and 83.55 ± 1.08% respectively. This increase among accuracies suggest the compound had a cardio-modulating effect on the cells at those given concentrations (n = 15). (**C**) Using the GCaMP6 method, significant changes in the beating rate were seen at the 30 nM concentration (p = 0.0175). (**D**) For the SR_90_ parameter, significant differences were detected at the 10 and 30 nM concentration (p = 0.0355 and p ≤ 10^−4^ respectively) (n = 15). *p < 0.05, ***p < 0.001.

E-4031 is a known antiarrhythmic drug that blocks the human Ether-à-go-go-Related (hERG) K^+^ channel and subsequently prolongs the QT interval^23^,^24^. The drug compound was administered to the iPS-CMs at an increasing concentration of 1, 3, 5, 10 and 30 nM. E-4031 induces QT prolongation, which was clearly seen in the contractile profiles generated from the brightfield images (Fig. 3a). At the 10 nM condition, extension of the relaxation phase was seen, consistent with the inhibition of the hERG K^+^ channel that contributes to the rapid repolarization of the cardiomyocytes. The lengthening of the relaxation phase became more evident and dramatic at the next treatment level, 30 nM.

For the brightfield method, the SVM accuracies for concentration levels of 1 nM, 3 nM, and 5 nM (47.38 ± 0.64, 50.45 ± 1.05, and 49.19 ± 1.49%) were similar to the baseline SVM accuracies (48.84%) (Fig. 3b). At the 10 nM concentration, the SVM was able to attain an accuracy of 79.82 ± 0.77%. The SVM accuracy continued to increase to 83.55 ± 1.08% at 30 nM concentration. For the GCaMP6 method, a change in beating rate was detected only at 30 nM with a 6.38% decrease (Fig. 3c). The SR_90_ increased by 10.73% and 21.51% for 10 nM and 30 nM respectively (Fig. 3d), which were statistically different from baseline.

### Response to verapamil

**Figure 4:**
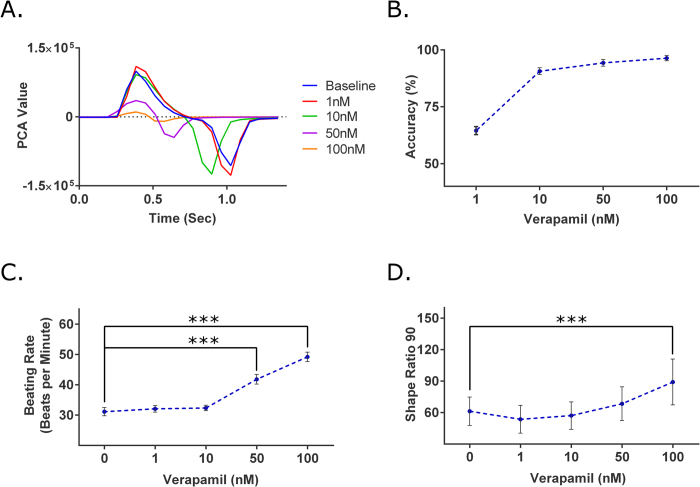
Analysis of verapamil, L-type Ca^2+^ channel blocker known to shorten QT duration. (**A**) Representative traces of the contractile profile from the brightfield analysis show the QT shortening effect begin at 10 nM and the negative inotropic effect at 50 nM. (**B**) At 1 nM, the SVM accuracy was 64.52 ± 1.78%. At the 10, 50, and 100 nM concentrations, the SVM accuracies were all above 90%, strongly indicating verapamil’s cardio-modulating effect. (n = 14). (**C**) In the GCaMP6 method, significant changes were detected at the 50 and 100 nM concentrations (p ≤ 10^−4^ and p ≤ 10^−4^). (**D**) Looking at SR_90_, significant differences were only detected at the 100 nM (p = 8.78 × 10^−4^) (n = 14). ***p ≤ 0.001.

Verapamil is an L-type Ca^2+^ channel blocker that is used therapeutically to treat cardiac arrhythmia^25^,^26^. The drug compound was administered at increasing concentrations of 1, 10, 50, and 100 nM. Verapamil causes QT shortening^27^, which was consistent with the observed decrease in the duration of the contractile profile, first seen at 10 nM (Fig. 4a). At 50 and 100 nM, the shortening effect became more pronounced. In addition, the amplitude of the contractile profiles significantly decreased, indicating a negative inotropic effect due to decreased levels of intracellular calcium. In the brightfield method, the 1 nM concentration level had a SVM accuracy value of 64.52 ± 1.78% (Fig. 4d). At the 10 nM level, the generated SVM models generated were able to achieve a SVM accuracy value that exceeded 90% (90.64 ± 1.49%). The SVM accuracy values continued to increase for the 50 nM and 100 nM concentration levels (94.32 ± 1.47 and 96.39 ± 1.16% respectively). With the GCaMP6 method, the beating rate decreased by 32.93% and 56.39% for 50 and 100 nM, respectively (Fig. 4c), which were statistically significant. A change in the SR_90_ value, a 45.42% increase, was statistically significant only at the highest concentration of 100 nM, (Fig. 4d).

### Response to blebbistatin

**Figure 5:**
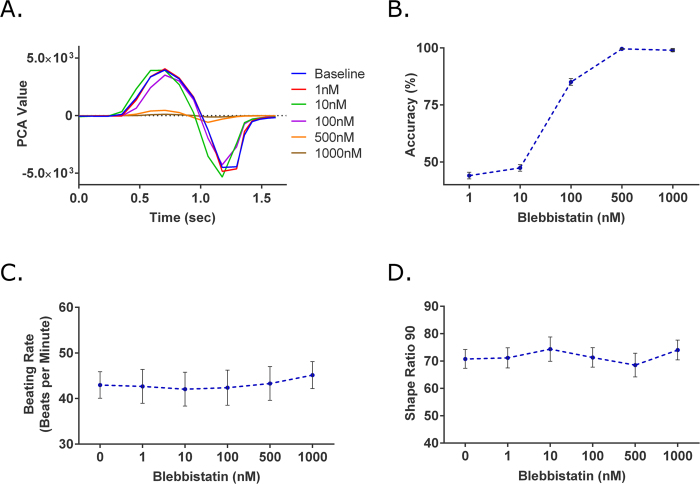
Analysis of blebbistatin, a myosin-II inhibitor and excitation-contraction decoupler. (**A**) Representative traces of the contractile profile from the brightfield method showed significant loss of contractility at higher concentrations. (**D**) At 1 and 10 nM the SVM accuracies were near 50%, suggesting minor to no changes in iPS-CMs. At 100 nM, the accuracy increased to 85.08 ± 1.49%. For 500 and 1000 nM, the respective SVM accuracies further increased and were both near 100% (n = 11). As expected with the GCaMP6 method, blebbistatin resulted in a false negative with no changes in (**C**) beating rate or (**D**) SR_90_ at any of the tested concentrations (n = 11).

Blebbistatin is a myosin-II inhibitor and a known excitation-contraction decoupler^28^. The drug compound was administered at an increasing concentration of 1, 10, 100, 500, and 1000 nM. The SVM accuracy values for the 1 nM and 10 nM concentration levels were near 50% (44.02 ± 1.45 and 47.38 ± 1.44% respectively) (Fig. 5d). However, at 100 nM, the SVM accuracy increased to 85.08 ± 1.49%. For both 500 and 1000 nM, SVM accuracy values increased to nearly 100% (99.67 ± 0.26 and 99.03 ± 0.57% respectively). Conversely, in the GCaMP6 method, there were no changes for any of the given concentrations (Fig. 5c,d).

As an excitation-contraction decoupler, blebbistatin induces a negative inotropic effect on the iPS-CMs. The expected negative inotropic effects were seen starting at 100 nM (Fig. 5a). At the highest concentration of 1000 nM, contraction and relaxation phases were still distinctly seen. However, the max amplitude of the contraction peak for the 1000 nM concentration was approximately 33-fold smaller than that of the baseline measurement (121.00 PCA value compared 3976.42 PCA value).

## Discussion

There is a pressing need for the development of new drug screening assays, as current methods remain inadequate at detecting and predicting the cardiotoxicity of compounds. Leveraging the capabilities of human stem cells to yield human cardiomyocytes, we present a simple and inexpensive method that can identify drug-induced cardiac effects in iPS-CMs. The method pairs brightfield imaging with optical flow and machine learning, and in all cases the method met or exceeded the sensitivity of a more traditional fluorescent-based intracellular calcium assay. Thus, we believe our method has the potential to be used in high-throughput assays to identify compounds with potential cardiotoxicity.

In conditions where no drug effects are anticipated (e.g. placebo experiments), the SVM accuracy values were expected to be around 50%, reflecting the random chance of correctly predicting the presence or absence of the drug or intervention. With successive addition of compounds that are cardioactive, we would expect an increase in the SVM accuracy as the decision boundary becomes more distinct. As a way to assess this method, iPS-CMs that are genetically encoded with a calcium indicator, GCaMP6, were used as a control method due to its similarities to multiple commercially available drug screening assays.

The average SVM accuracies obtained from the three longitudinal time points (90, 120, and 150 minutes) was 48.84%, and the average of all four placebo conditions was 50.42%. Furthermore, the GCaMP6 data had no statistical significance in the recorded parameters among the conditions in both these control experiments. This data suggests only small and negligible, variations in iPS-CM behavior over time. Thus, both the longitudinal and placebo experiments confirmed that when machine learning is applied to data of iPS-CMs in the absence of cardioactive compounds, the SVM accuracy values would be in the range of 50%.

For the drug experiments, all three compounds were purposely selected for their known effect on iPS-CM behavior. The compounds E-4031 and verapamil are known to have very strong electrophysiological effects and only secondarily affect contractility. While calcium transients are not a direct readout for cardiomyocyte electrophysiology, previous researchers have shown that changes in calcium transient are reliable surrogate readouts for changes in electrophysiology as they reflect changes in the excitation-contraction coupling^29^,^30^,^31^. Assays that utilize calcium transient measurements have been shown to specifically detect changes in human stem cell-derived cardiomyocytes induced by E-4031 and verapamil^29^,^31^. By demonstrating that the brightfield method can capture the effects of these drugs (E-4031 and verapmil) that primarily affect cardiomyocyte electrophysiology, we will have shown that the brightfield method is not only effective to screen compounds that primarily affect contractility but is more broadly applicable.

As expected, a more pronounced difference in iPS-CM behavior caused by increasing concentrations of cardioactive compounds resulted in an increase in accuracy of the machine’s prediction, as summarized in Table 1. Looking at E-4031, the SVM accuracy values remained around 50% at the lower concentrations (1, 3, and 5 nM), indicating that there were no cardioactive effects at the given concentrations. As higher concentrations were introduced, noticeably higher SVM accuracy values were achieved (79.82% and 83.56% for 10 and 30 nM respectively), suggesting drug-induced effects. These observations were corroborated by the GCaMP6 data, which showed statistical significance among the parameters at 10 and 30 nM. This separation in accuracy values highlights the utility and applicability of machine learning for drug screening purposes.

Interestingly, at 10 nM of verapamil, a SVM accuracy of 90.64% was reported, indicating a change in cardiomyocyte behavior, while the lowest concentration level that had a significant change in the GCaMP6 data was at 50 nM. Verapamil inhibits the L-type Ca^2+^ channel, thus reducing both the amplitude and duration of the calcium transient. Thus, when normalizing by the amplitude to calculate SR_90_, the decrease in CTD_90_ could be masked by the decrease in amplitude. The shortened contractile profile (Fig. 4c) and decrease in CTD_90_, may suggest that the effects of verapamil were masked at lower concentrations. If this is the case, the increases in SR_90_ values at higher concentrations indicate that the relative decrease in the amplitude is greater than the decrease in the duration of the calcium transient.

The SR_90_ parameter is prone to inaccuracies in drug compounds that elicit similar and simultaneous changes in the amplitude and duration of calcium transients. However it is necessary as a means of normalization to counteract the photobleaching effect seen in the GCaMP6 method. Attempts to normalize the decay to the control experiments were not possible as the decay pattern among each sample of the longitudinal experiments greatly differed (Supplemental Fig. 2). Due to unpredictability of the this decay, modeling the decreasing trend as linear or exponential functions dependent on the acquisition number was unsuccessful as well. This photobleaching effect is not unique to the GCaMP6 method, but rather present in most fluorescence-based techniques. Although there are ways to minimize photobleaching effects, there are considerable trade-offs. One method of adjustment is reducing the laser intensity; however, this reduces signal-to-noise ratio and does not remove the photobleaching effect altogether. A line-scan mode of acquisition can significantly reduce photobleaching, but this method eliminates wide field-of-view image acquisition, making it less suitable for high-throughput screening. The brightfield method is not subject to this photobleaching effect and can be adapted for high–throughput, longitudinal studies on the same set of the iPS-CMs without degradation of signals.

Blebbistatin, a myosin II inhibitor, was expected to have no changes in the GCaMP6 signal as the inhibition occurs downstream in the proteins that generate the force of contraction, and not intracellular calcium. The predictions of blebbistatin as a false negative in the GCaMP6 method were confirmed as the analysis showed there were no differences in any of the tested concentrations. However, in the brighfield method, a decrease in the amplitude of the contractile profile was seen starting at 100 nM. This experiment clearly demonstrates that methods based on the electrophysiological profiles, such as patch clamping, fluorescence-based optics, and MEA, are prone to type II errors with drugs such as blebbistatin that decouple excitation and contraction of iPS-CM. Thus, it is crucial in future cardiotoxicity drug screening assays to have a component that monitors contraction and relaxation of the iPS-CMs.

The progression of including assays that monitor contractility is supported by the number of commercially available systems that use impedance-based arrays^18^,^32^,^33^,^34^,^35^. However, impedance-based arrays require a minimum number of iPS-CMs in a monolayer to attain a signal. Because various culture techniques have been presented to acquire more physiologically relevant iPS-CMs including multi-culture systems with fibroblasts and endothelial cells, pacing, and culturing on 3-D platforms with anisotropic features, the implementation of impedance-based arrays with such techniques is very difficult^36^,^37^,^38^,^39^,^40^,^41^. In contrast, the brightfield method is simple as the only requirement is an optically clear platform that allows real-time acquisition of brightfield images. The flexibility also allows this method to complement current techniques, such as intracellular calcium transients, to provide better accuracy. Furthermore, this method is not limited to high-throughput drug screening purposes; it can be easily employed for basic research use (e.g. as a readout in the study of iPS-CM maturation).

The concept of using image-based analysis of cardiomyocyte beating dynamics has been explored by various research groups^42^,^43^,^44^. However, these studies have all required the need for custom cell culture apparatuses. More recently, research groups have investigated brightfield-based methods that are widely applicable to both standard cell culture apparatuses, such as a 384-well cell culture plates, and custom ones^45^,^46^,^47^,^48^,^49^,^50^,^51^,^52^. These respective methods have all demonstrated the feasibility of tracking the contractile behavior of cardiomyocytes and extracting out quantitative parameters to describe the apparent motion. Maddah *et al*. validated the practicability of using brightfield microscropy to monitor contractile behavior of cardiomyocytes in various setups: monolayer, cardiosphere and single cell^53^. There are primarily two strategies in achieving this image-based analysis: (i) methods that derive signals based on pixel differences or derivatives between frames and (ii) methods that derive signals based on generated motion vectors.

In this study, the latter, a vector-based strategy using optical flow, was chosen as we have previously demonstrated that the directional component of a vector can be crucial in the analysis of cardiomyocyte motion^19^. When looking at just the magnitude of pixel differences or vector displacement, a contractile beat is assumed to be represented by two distinct peaks (one representing the contraction phase and one representing relaxation)^49^,^51^. The ability to discern between the two phases can be achieved if a resting phase is present. However, if the beating frequency of the cells is increased, multiple phases can be embedded into one singular peak as we have shown in our previous study^19^. Thus, methods that analyze both the magnitude and directional components of vectors should provide more detailed information.

Admittedly, a potential limitation in this study’s set-up is the higher computational resource requirement of the used optical flow algorithm when compared to other algorithms used for vector-based methods (e.g. block-matching and minimum quadratic difference algorithms)^47^,^50^,^51^,^52^. However, the advantages of image segmentation and signal processing using PCA along with machine learning analysis can be leveraged by applying these techniques to the vectors of such other algorithms. The implementation of PCA summarizes the magnitude and directional components of vectors into one dimensional data. It automatically discerns the contraction phases (positive PCA values) from the relaxation phases (negative PCA values). In addition, while not demonstrated in this study, the calculated spatial PCA (Supplementary Information) can be applied to segment asynchronously beating cell clusters. The SVM accuracy calculated in our method serves as a singular quantitative index or metric that summarizes not only the impact of the 12 parameters, but also any underlying relationships or interactions among parameters. This index becomes valuable for high-throughput screening of drug-induced cardiotoxicity by highlighting compounds that have any cardioactive effect on the contractile behavior of cardiomyocytes.

In summary, we present a screening method that combines brightfield microscopy and machine learning to enable the sensitive detection of changes in the contraction of human iPS-derived cardiomyocytes. Unlike fluorescence-based methods that suffer from photobleaching, this brightfield method does not result in the loss of signal over time even in longitudinal studies. Furthermore, this approach can be implemented in combination with other screening methods, and provides insights on how patterns in electrophysiological data are coupled to those of contraction. Moreover, this method is easily adaptable to various configurations, even 3D tissue models, to enable easy, robust, and information-rich readouts of cardiomyocyte behavior.

## Methods

### Cell culture

A human iPS cell line constitutively expressing the calcium sensitive fluorescent protein GCaMP6 (WTC11-AAV-CAG-GCaMP6) was obtained from Dr. Bruce Conklin (Gladstone Institute of Cardiovascular Research). The cells were grown on 6-well plates coated with Matrigel (Corning) in mTeSR1 (Stem Cell Technologies) with daily media replacement. The cells were passaged at 80% confluence using StemPro Accutase (Life Technologies) and seeded on Matrigel-coated plates in mTeSR1 containing 10 μM Y-27632 (Tocris), a Rho-associated protein kinase (ROCK) inhibitor. A small molecule Wnt modulatory protocol was used for cardiomyocyte differentiation^54^. In brief, the cells were seeded onto Matrigel-coated 12-well plates at 100,000 cells/well, and the cells are grown for 3 days prior to differentiation. On Day 0, the media was switched to RPMI 1640 (Life Technologies) and B-27 supplement without insulin (RPMI/B-27-Ins, Life Technologies) containing 12 μM CHIR99021 (Selleckchem), a glycogen synthase kinase 3 (GSK3) inhibitor. On Day 1, the media was replaced by RPMI/B-27-Ins. On Day 3, the media was replaced by RPMI/B-27-Ins containing 5 μM IWP2 (Tocris), a Wnt signaling inhibitor. On Day 5, the media was replaced by RPMI/B-27-Ins. On Day 7, the media was replaced by RPMI/B-27 followed by media replacement every 3 days. Spontaneously contracting cells were observed between Day 10 to 13. After 20 days post-differentiation, cardiomyocytes were purified using lactate selection^55^. In brief, the cells are washed three times using DPBS (Life Technologies), and the media was replaced by glucose-free DMEM (Life Technologies) containing 4 mM lactic acid (Sigma-Aldrich) and 25 mM HEPES (Life Technologies). The media was replaced every 2 days for 8 days, with gentle shaking to dislodge dead cells.

### Drug Treatment

Cardiomyocytes were passaged by incubating in collagenase II (Life Technologies) for 1 hour followed by TrypLE Select (Life Technologies) for 4 minutes. The cells were counted and seeded as a dense monolayer on Matrigel-coated 8-chamber Lab-Tek II Chamber Slides (Electron Microscopy Sciences) at 150,000 cells/well. The drugs E-4031, verapamil, and blebbistatin (Sigma-Aldrich) were delivered in RPMI/B-27 at various concentrations known to have cardioactive effects^16^. Prior to exposure to drug compounds, three baseline measurements were made at time points 0, 30, and 60 minutes. Drugs were serially added to the wells, waiting 20 minutes after addition before taking measurement. RPMI/B-27 was used as placebo. The drug studies were performed on cardiomyocytes at 40+ days post-differentiation.

### Time Lapse Imaging of Cardiomyocyte Motion

Images of contracting cardiomyocytes were acquired with an Olympus IX83 (Olympus) inverted scope equipped with an ORCA-R2 color charge-coupled (Hamamatsu) camera and MetaMorph software (Molecular Devices). To control for temperature and pH, an incubation system (model ZILCS) consisting of a stage top incubator, temperature controller, and gas flowmeter (Tokai Hit) was used with 5% CO_2_. Both fluorescent and brightfield acquisitions were taken for approximately 17.5 seconds at 15.6 fps at a resolution of 672 × 512 or at 8.6 fps at a resolution of 1392 × 1040 (subsequently downsized to a resolution of 672 × 512 via bilinear interpolation) and saved as a TIFF stack. A script in MetaMorph was created to acquire the fluorescent image automatically after the brightfield acquisition was completed. To address variation among cell populations within the same sample, stage locations were recorded prior to any acquisitions to ensure that the same cells among a sample were imaged.

### Intracellular Calcium from the GCaMP6 Fluorescence

The TIFF stacks were imported to ImageJ (NIH), where a 240 by 240 pixels region of interest (ROI) were selected for each stack. The average intensity value within the ROI for each frame was measured and imported into MATLAB (MathWorks). An automated code was written to identify the start and the peak of each contraction. An *n*-th order (*n* = 2–10) polynomial fit was used to normalize the decay of signal due to photobleaching. The data was used to calculate the beating rate, peak amplitude, and CTD_90_. From this, the ratio of the CTD_90_ and the amplitude was calculated, and defined as the shape ratio (SR_90_). The SR_90_ is relatively insensitive to photobleaching (both numerator and denominator impacted equally), and was thus used to correct for photobleaching of signal over time.

### Optical Flow & Machine Learning

Brightfield images were processed with an optical flow algorithm, as previously described, to generate matrices of x- and y-directional vectors that represent cardiomyocyte motion^19^,^56^. Using a custom MATLAB script, principal component analysis (PCA) was used to summarize the magnitude and directionality of the vectors into a single variable (Supplemental Information). When this single variable was plotted over time, a contractile profile was generated. These contractile profiles have distinct peaks that represent the contraction and relaxation. With the contractile profiles, the user was prompted to identify the start of the first contraction for each recording. By doing so, peaks with positive principal component analysis (PCA) values were identified as the contraction phase and those with negative PCA values as the relaxation phase of the cardiomyocyte beating motion. The software then calculates 12 parameters that describe the overall shape of the contractile profiles (e.g. duration of the contraction phase) (Supplemental Fig. 1). The frequency parameter was defined as the inverse of the time (seconds) between the peak of the contraction phase for a given beat and that of a subsequent beat. Based on this definition, the frequency parameter can not be calculated for the last beat acquired. Therefore any other calculated parameters associated with the last beat were removed from the machine learning portion.

Each of the cardiomyocyte contractions was then regarded as an individual sample for the machine learning (ML) analysis. Support vector machine (SVM), a classifying ML algorithm, was implemented to discern between normal and abnormal cardiomyocyte contractile profiles. As most biological data do not have linear trends, a non-linear kernel, radial basis function, was chosen for SVM. The classification between the contractions of the baseline measurements and those of a given drug concentration was accomplished by randomly separating the data into a training set and a test set. The data in the test set was withheld from the training process and only used to evaluate the model. The two sets were formed by first randomly grouping one third of the wells and the corresponding contractions of that drug condition into the test set. To ensure that the test set has equal number of baseline and drug samples, the baseline measurements for one ninth of the wells were randomly allocated for the test set. The reason was that the number of baseline measurements for each well outnumbered each drug condition of that corresponding well three to one. The rest of the wells and respective contractions were used as the training set. Within the training set, a 5-fold cross validation was performed to optimize the parameters for the generated SVM model. The optimized SVM model was then used to classify the test set. The SVM accuracy of the model was calculated by the ratio of correct classifications to the total classifications for the test set. To account for potential sampling bias, this process was performed 50 times and the reported SVM accuracy was the average of all the runs. With this process repeating numerous times and having random allocation each time for the test and training sets, the size of each respective set varied from run to run. For the baseline measurements, each well had approximately 9 contractions per video acquisition. With these numbers of contractions, the training set used for the SVM classifier had at least 200 samples.

### Statistics

Statistical significance of GCaMP6 data was determined with a one-way repeated measure ANOVA. A key assumption made in repeated measures ANOVA was the condition of sphericity, the variance of the differences among all possible pairs of a group is equal. Sphericity was determined using Maulchy’s Test for Sphericity (p-value <0.05). If the assumption of sphericity was not correct, the Greenhouse-Geisser correction was used to adjust the reported p-values for the one-way repeated measures ANOVA. If a significant p-value (p-value <0.05) was obtained from the Repeated-Measures ANOVA, we proceeded with Dunnett’s test to determine which concentration differed from our baseline measurements. Differences with p-values less than 0.05 were considered statistically significant. All reported values are in the format of mean + S.E.M. All reported sample sizes (n) refer to independent wells of chamber slides (biological replicates).

## Additional Information

**How to cite this article**: Lee, E. K. *et al*. Machine learning plus optical flow: a simple and sensitive method to detect cardioactive drugs. *Sci. Rep*. **5**, 11817; doi: 10.1038/srep11817 (2015).

## Supplementary Material

Supplementary Information

## Figures and Tables

**Table 1:** Summary of the SVM accuracies calculated for the concentrations of each drug compound with the brightfield method.

E-4031		Verapamil		Blebbistatin	
Concentration	Accuracy	Concentration	Accuracy	Concentration	Accuracy
1 nM	47.38%	1 nM	64.52%	1 nM	44.02%
3 nM	50.45%	10 nM	90.64%	10 nM	47.38%
5 nM	49.19%	50 nM	94.32%	100 nM	85.08%
10 nM	79.82%	100 nM	96.38%	500 nM	99.63%
30 nM	83.55%			1000 nM	99.04%
