# Supplementary material for: Machine learning plus optical flow: a simple and sensitive method to detect cardioactive drugs
Source: Sci Rep. 2015 Jul 3;5:11817. doi: 10.1038/srep11817 (PMC4490343; doi:10.1038/srep11817)
Supplement: Supplementary Information [file srep11817-s1.pdf]

Machine Learning plus Optical flow: A simple and sensitive method to detect cardioactive drugs

Eugene K. Lee[1,#], Yosuke K. Kurokawa[2,#], Robin Tu[3], Steven C. George[2,4,§], and Michelle Khine[1,5,§,*]

Department of Biomedical Engineering[1], University of California, Irvine, Irvine, CA 92697.

Department of Biomedical Engineering[2], Washington University in St. Louis, St. Louis, MO 63130.

Department of Statistics[3], California Polytechnic State University, San Luis Obispo, San Luis Obispo, CA 93410.

Department of Energy, Environment, and Chemical Engineering[4], Washington University in St. Louis, St. Louis, MO 63130.

Department of Chemical Engineering and Material Science[5], University of California, Irvine, Irvine, CA 92697.

[#,§] Authors contributed equally to this work
[*] Corresponding Author
Correspondence to: Michelle Khine (mkhine@uci.edu)

Supplementary Information

Supplementary Methods

Generation of Contractile Profile

After the image is processed with the optical flow algorithm, a matrix containing the magnitude of the x- and y-vectors throughout the acquisition period will be generated. A mask is then created to identify and segment out the region of interest. This task is done by performing a PCA analysis with an eigendecomposition for spatial resolution. The norms of the eigenvalues of the 1st PCA are then sorted in order of highest to lowest. The lowest number in the first quartile is then regarded as the mask threshold. Using this mask threshold, a spatial mask is generated to describe regions that have generated the most motion throughout the acquisition. With the regions of interest identified, a PCA is done on the selected area for temporal resolution. When the norm of these eigenvalues are plotted out, the contractile profile is generated.

Supplementary Figures

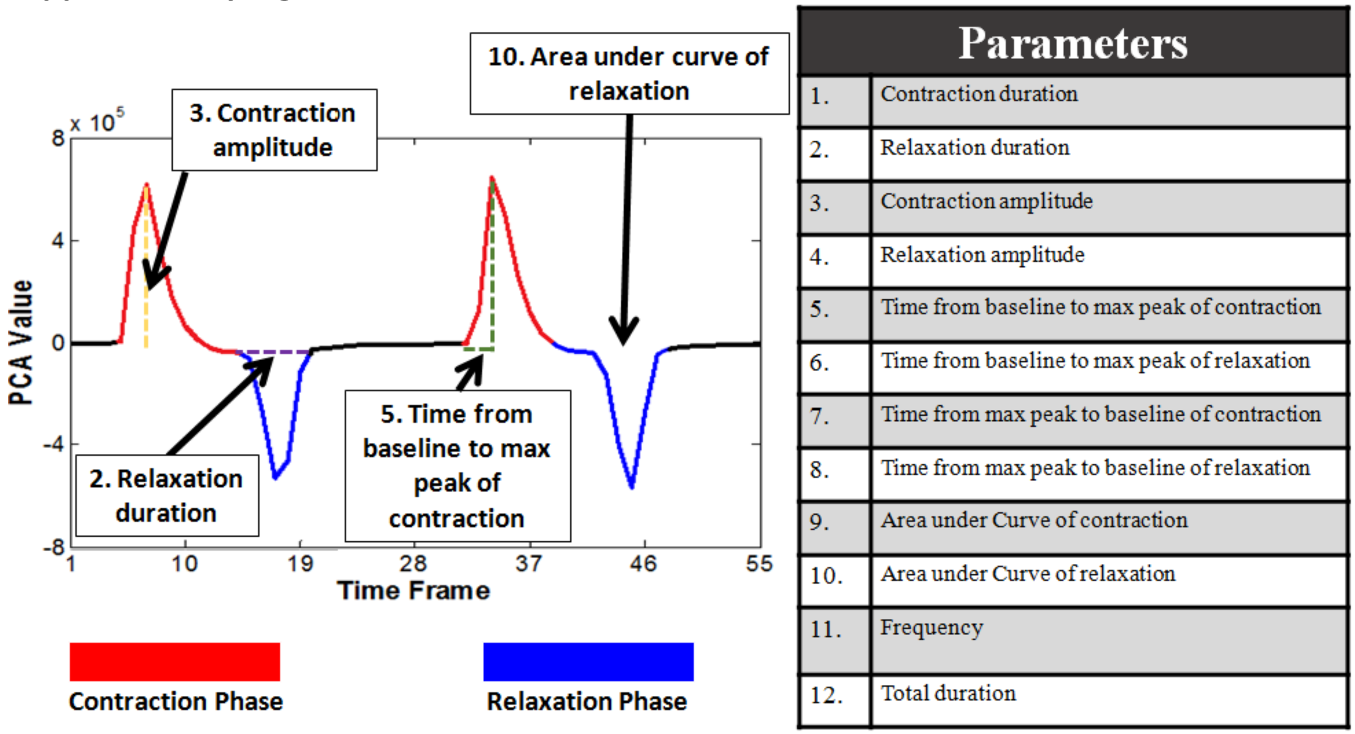

Parameters	
1.	Contraction duration
2.	Relaxation duration
3.	Contraction amplitude
4.	Relaxation amplitude
5.	Time from baseline to max peak of contraction
6.	Time from baseline to max peak of relaxation
7.	Time from max peak to baseline of contraction
8.	Time from max peak to baseline of relaxation
9.	Area under Curve of contraction
10.	Area under Curve of relaxation
11.	Frequency
12.	Total duration

Supplementary Figure 1: Parameters derived from the contractile profiles based on the 1st PCA of motion vectors. (Left panel) Red indicates the contraction phase of an iPS-CM beat and has positive PCA values. The blue indicates the subsequent relaxation phase of a beat and is designated by negative PCA values. (Right panel) List of the 12 parameters that are extracted from the contractile profile.

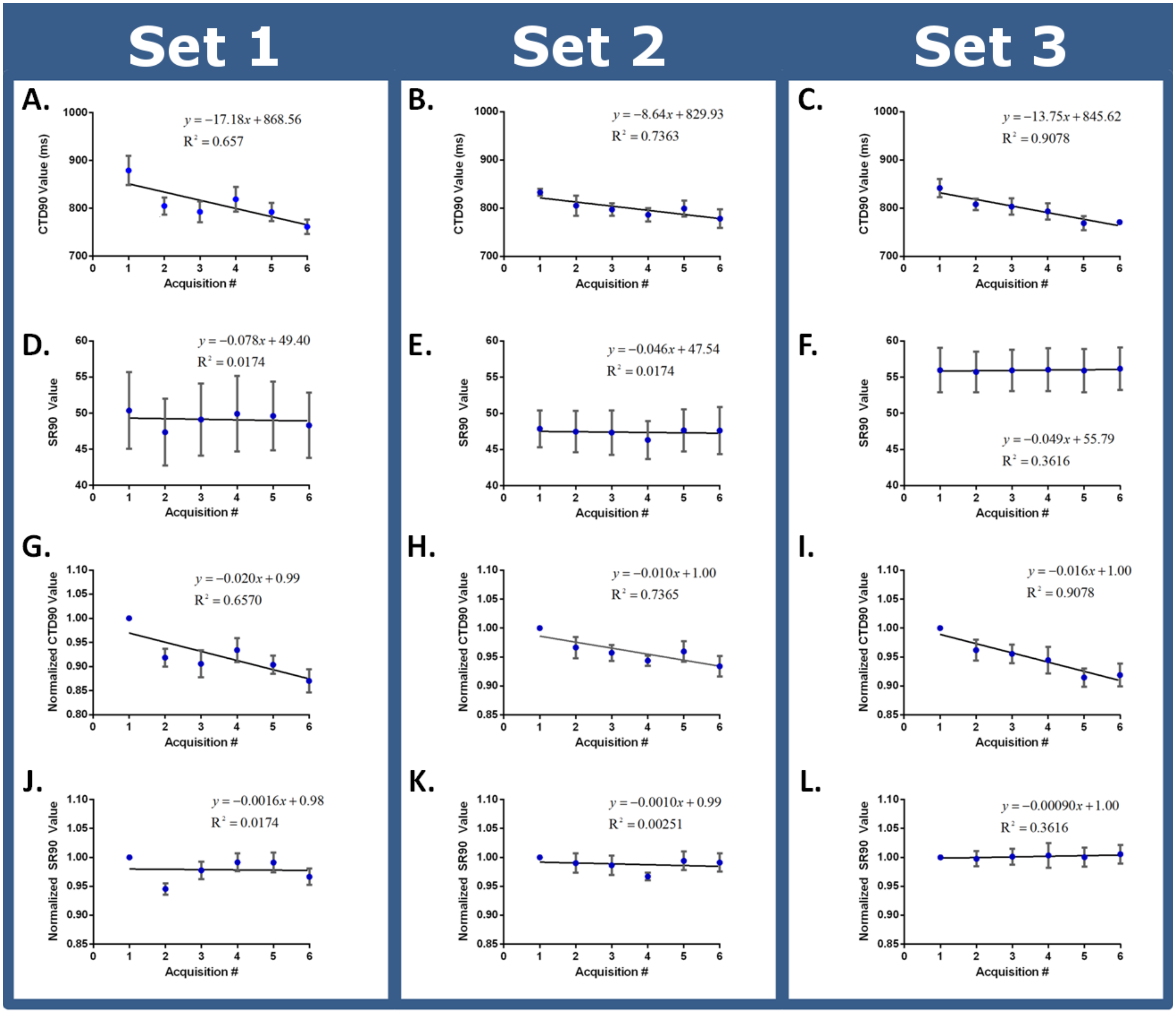

Supplementary Figure 2: (A-C) Set 1 &2 of the longitudinal experiments had acquisitions taken at intervals of 30 minutes; while Set 3 had acquisitions taken at intervals of 9 minutes with exception of last time point (time point was taken at 90 minutes instead). The results showed a similar decrease in CTD_{90}, suggesting that there is little to no recovery from photobleaching during the length of the experiment. While the decrease is similar, the CTD90 values among each of the sets of microscope chamber slides used for the longitudinal experiment decreased at different rates. (D-E) The calculated SR90 for all three sets removed the decaying trend among acquisitions. (F-G) To control for the different initial CTD90 values, all subsequent values were normalized to the starting values. The normalization still yielded decreasing trends that differed among the sets. (J-L) The best-fit lines generated from the linear regression of the normalized SR90 values were relatively uniform.

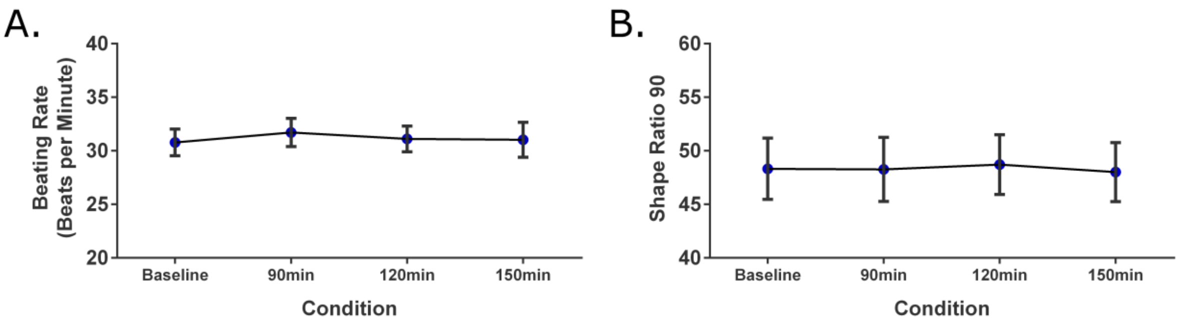

Supplemental Figure 3: Analysis of GCaMP6 data from longitudinal study. With the GCaMP6 method, no significant changes in the (A) beating rate or (B) SR_{90} were detected at any of the time points (n = 13).

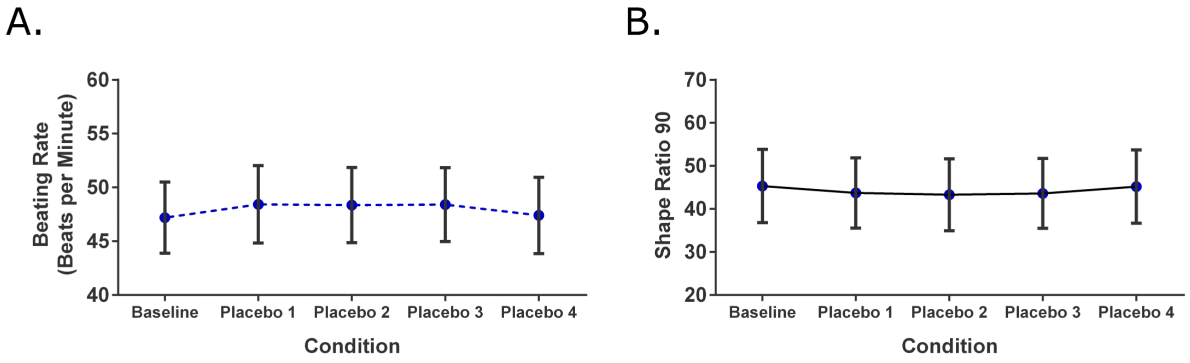

Supplemental Figure 4: Analysis of GCaMP6 data from placebo study. During the length of the placebo experiments, there was no observable change in the (A) beating rate or (B) SR_{90} using the GCaMP analysis (n =18).
